# S39. GPR139 AN OPHAN GPCR AFFECTING NEGATIVE DOMAINS OF SCHIZOPHRENIA

**DOI:** 10.1093/schbul/sby018.826

**Published:** 2018-04-01

**Authors:** Joy Atienza, Holly Reichard, Victoria Mulligan, Jackie Cilia, Holger Monenschein, Deanna Collia, Jim Ray, Gavin Kilpatrick, Nicola Brice, Mark Carlton, Steve Hitchcock, Ged Corbett

**Affiliations:** Takeda

## Abstract

**Background:**

Individuals with schizophrenia fail to appropriately use negative feedback to guide learning. These learning deficits are thought to arise from abnormalities in midbrain dopamine activity. The habenula is a well conserved paired structure that sits in the midline, adjacent to the third ventricle, and dorsal and posterior to the thalamus. Classic studies have shown that it is part of the reward pathway and functions with dopamine neurons in the ventral tegmental area (VTA) to mediate reward related signals, specifically aversive and negative stimulus (Hikosaka, 2010). Several studies point to pathology in the habenula as contributing to schizophrenia (Sandyk, 1992; Caputo et al., 1998; Shepard et al., 2006). Despite the many studies on the habenula, the precise function of the habenula remains unclear. Here we describe functional consequences of regulation of GPR139 an orphan GPCR that is specifically expressed in the CNS and enriched in the habenula (Matsuo et al., 2005) in mouse models of domain of schizophrenia.

**Methods:**

Specific expression of mouse GPR139 in the habenula was evaluated using bacterial artificial chromosome translating ribosome affinity purification (bacTRAP) and confirmed by immunohistochemistry. GPR139-/- mice were generated by removal of a 736bp region encoding the seven transmembrane domain (7TM) domain. Knockout animals were maintained inbred on a 129/SvEv background and evaluated in behavioral models that reflect aspects of negative symptoms. High throughput screen (HTS) for small molecules was performed. We expressed full length GPR139 into CHO cells and screened a 600K library using a calcium assay to identify small molecule agonist. Hits were identified and physical properties optimized to produce a molecule suitable for in vivo evaluation. GPR139 agonist was testedin vivo in social interaction in Poly(I:C) and cognitive test in subchronic PCP, models of schizophrenia.

**Results:**

Using bacTRAP we observed enriched expression of GPR139 in substance P positive- cells of the medial habenula. This expression was confirmed with immunohistochemistry. To determine if GPR139 regulates the known role of the habenula in learning, motivation, and social behaviour, GPR139 -/- animals were generated and phenotyped. These animals appeared normal and performed comparably to wild-type animals in a range of standard tasks. However, they were significantly impaired in models that reflect aspects of negative symptoms such as progressive ratio (Killeen et al., 2009; Heath et al., 2016), a measure of motivation and nest-building (Pedersen et al., 2014; Nakajima et al., 2013), a model of self-neglect. Furthermore, these animals showed deficits in novel object recognition model of working memory (Antunes and Biala, 2012). The small molecule agonist was observed to reverse deficits in models of schizophrenia including cognition in a subchronic PCP induced attentional set-shifting paradigm (Birrell and Brown, 2000) and social interaction in the Poly(I:C) maternal model (Meyer et al., 2008; Bitanihirwe et al., 2010).

**Discussion:**

These findings identify an orphan receptor that plays an important role in habenular function. Modulation of this receptor may provide an opportunity to address an important unmet need in schizophrenia.

